# Supplementary material for: Phospho-Mutant Activity Assays Provide Evidence for the Negative Regulation of Transcriptional Regulator PRE1 by Phosphorylation
Source: Int J Mol Sci. 2020 Dec 2;21(23):9183. doi: 10.3390/ijms21239183 (PMC7729563; doi:10.3390/ijms21239183)
Supplement: Supplementary file 1 [file ijms-21-09183-s001.pdf]

A

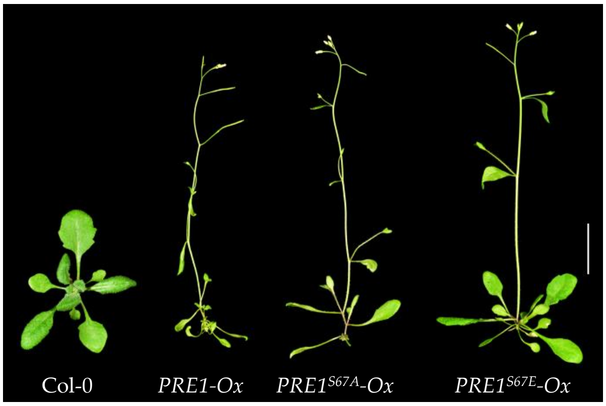

B

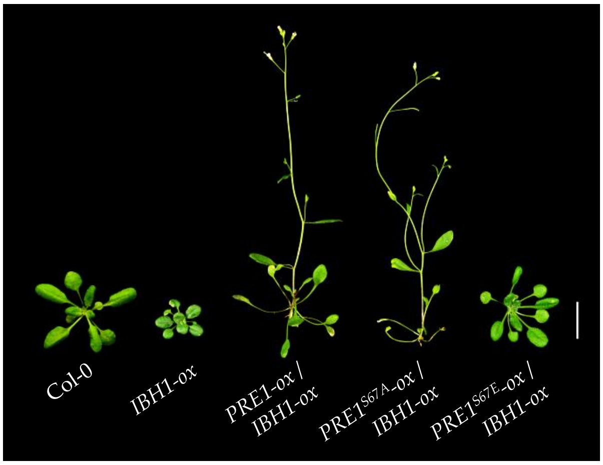

Figure S1. Phospho-mimicking mutation of Ser-67 to Glu-67 of PRE1 impaired its function and failed to suppress the dwarf phenotypes of *IBH1-Ox*. (A) Phenotype of Wild type Col-0 and transgenic plants expressing PRE1 and mutated PRE1 grown in soil for 4 weeks under long-day condition. Scale bar=20 mm. (B) Phenotype of wild type Col-0, *IBH1-Ox, IBH1-Ox/PRE1-Ox*, *IBH1-Ox/PRE1^{S67A}-Ox* and *IBH1-Ox/PRE1^{S67E}-Ox* plants grown in soil for 4 weeks under long-day condition. Scale bar=10 mm.

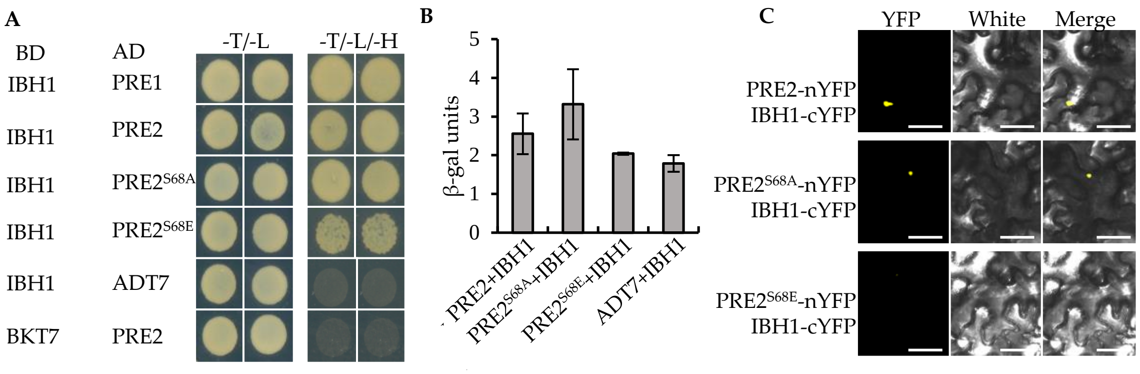

Figure S2. PRE2^{S68E} reduced the binding affinity to IBH1 in vivo and in vitro. (A) Yeast two-hybrid assays showed that mutation of Ser-68 of PRE2 to Glu-68 reduced the interaction of PRE2 with IBH1. (B) Quantitative analysis the β-galactosidase expression which induced by interaction between BD-IBH and various versions of AD-PRE2 in the yeast. Error bars indicated S.D. from three biological repeats. (C) BiFC assays showed that IBH1 interacted with PRE2, PRE2^{S68A}, but not PRE2^{S68E} in tobacco leaves. Scale bars=20 μm.

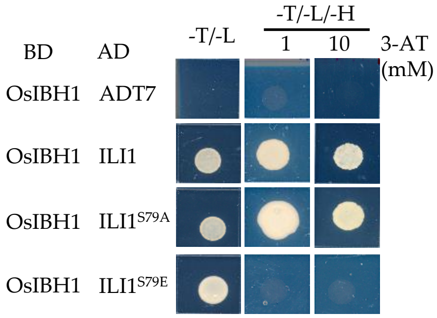

Figure S3. OsIBH1 interacted with ILI1 and ILI1^{S79A}, but not ILI1^{S79E} in yeast. Yeast two-hybrid assays showed that mutation of Ser-79 of ILI1 to Glu-79 reduced the interaction of ILI1 with OsIBH1.

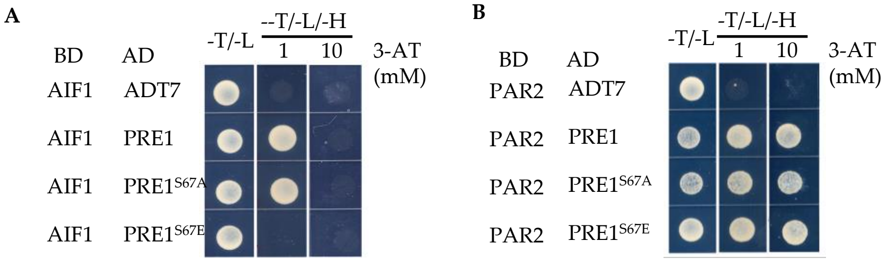

Figure S4. Phospho-mimicking mutation of Ser-67 to Glu-67 resulted in the weak binding ability of mPRE1 to its partners AIF1 and PAR2. (A) and (B) Yeast two-hybrid assays showed that mutation of Ser-67 of PRE1 to Glu-67 reduced the interaction of PRE1 with AIF1 and PAR2.

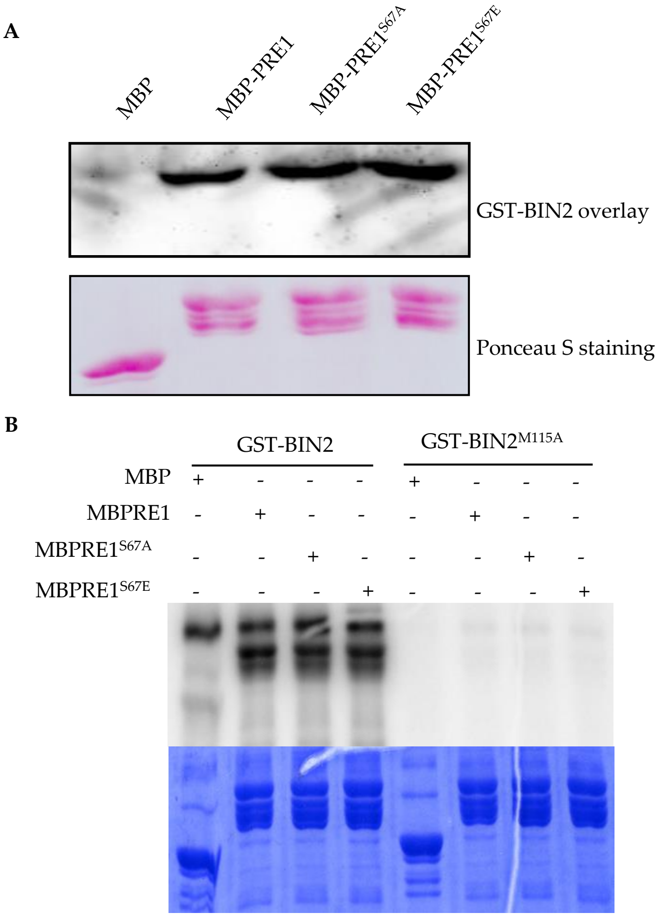

Figure S5. BIN2 interacted with and phosphorylated PRE1, PRE1^{S67A} and PRE1^{S67E} in vitro. (A) A gel blot of MBP, MBP-PRE1, MBP-PRE1^{S67A} and MBP-PRE1^{S67E} was probed with GST-BIN2 followed by horseradish peroxidase-labeled anti-GST antibody or stained with Ponceau S (Stain). (B) In vitro kinase assay showed that GST-BIN2 phosphorylated MBP-PRE1, MBP-PRE1^{S67A} and MBP-PRE1^{S67E}, but not MBP only. GST-BIN2 or kinase dead GST-BIN2^{M115A} were incubated with MBP, MBP-PRE1, MBP-PRE1^{S67A}, MBP-PRE1^{S67E} and 32P-γATP for 3 hrs. at 30°C. CBB indicates Coomassie brilliant blue stained-gel.

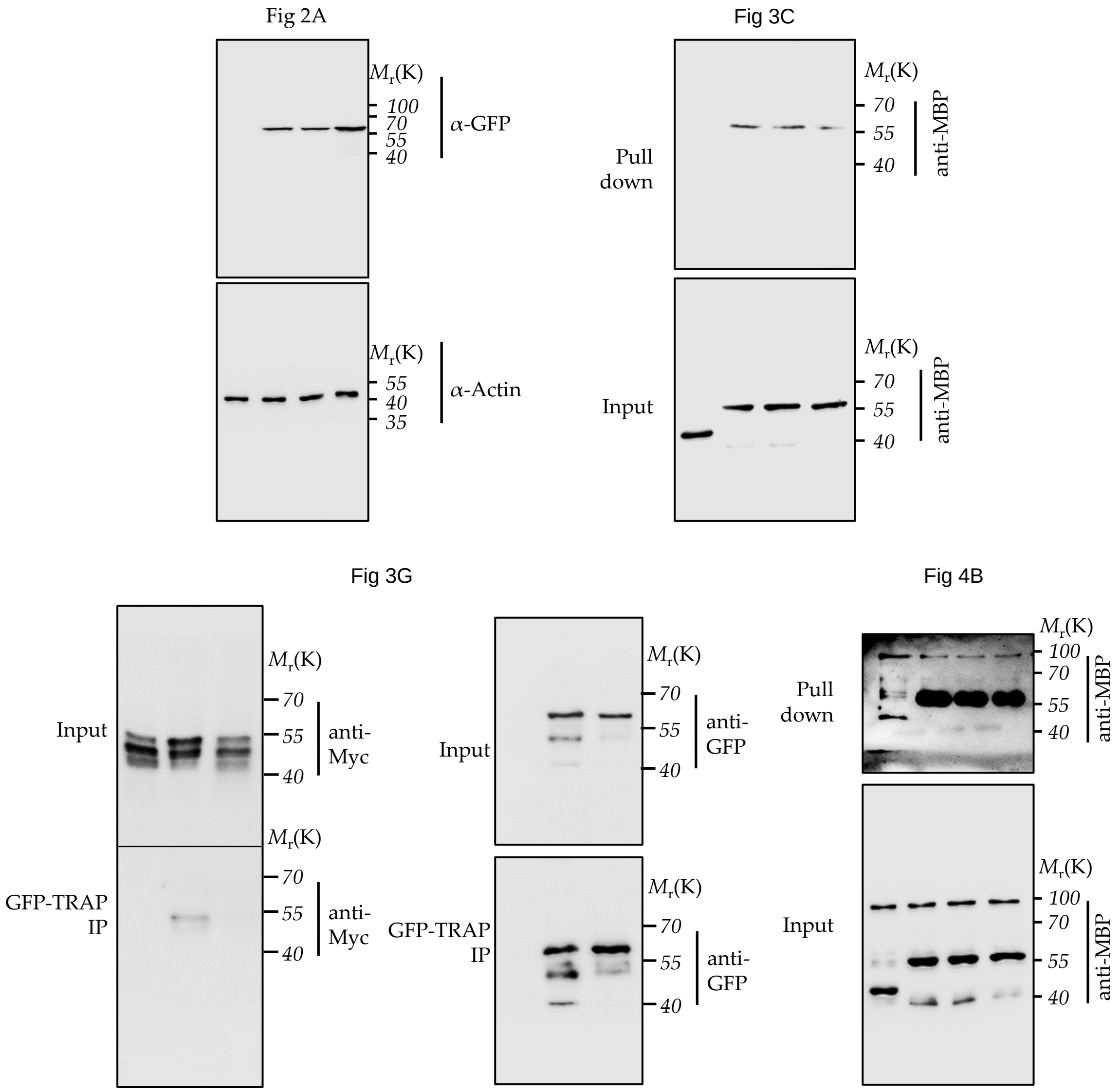

Figure S6. Uncropped image of immunoblots.
